# Development of insulin and its pharmacology and perioperative use: a narrative review

**DOI:** 10.1016/j.bja.2025.05.006

**Published:** 2025-06-06

**Authors:** Ketan Dhatariya, Nicholas A. Levy, Daniel Stubbs, Claire Frank, Sarah L. Tinsley, Roger D. Knaggs

**Affiliations:** 1 Elsie Bertram Diabetes Centre, Norfolk and Norwich University Hospitals NHS Foundation Trust, Norwich, UK; 2 University of East Anglia Medical School, Norwich, UK; 3 Department of Anaesthesia and Perioperative Medicine, West Suffolk NHS Foundation Trust, Bury St Edmunds, UK; 4 Department of Anaesthesia, Cambridge University Hospitals NHS Foundation Trust, Cambridge, UK; 5 National Institute for Health and Care Research (NIHR) Advanced Fellow, Perioperative, Acute, Critical, and Emergency Care Section, University of Cambridge, Cambridge, UK; 6 Pharmacy Department, Wrexham Maelor Hospital, Betsi Cadwaladr University Health Board, Wrexham, UK; 7 Centre for Perioperative Care, Royal College of Anaesthetists, London, UK; 8 Pharmacy Department, University Hospitals of North Midlands, Stoke-on-Trent, UK; 9 School of Pharmacy, Pain Centre Versus Arthritis, University of Nottingham, Nottingham, UK

**Keywords:** continuous glucose monitoring, diabetes mellitus, hybrid closed loop, insulin, insulin infusion, insulin pump, perioperative

## Abstract

Diabetes mellitus is characterised by an elevated blood glucose concentration. Over the last two decades, a plethora of new agents have emerged to help treat the condition, of which several classes of agent have been shown to reduce the risk of cardiovascular morbidity and mortality. In addition, there have been several developments in the pharmacology of insulin, improving the pharmacokinetics and pharmacodynamics of insulin analogues to better mimic physiological insulin concentrations in the liver, skeletal muscle, and other tissues. Furthermore, the technologies used to deliver insulin and measure glucose have improved; for example, in the UK, hybrid closed loop systems are now the standard of care for people with type 1 diabetes mellitus. This review focuses on insulin and insulin delivery. We consider the history of insulin development and the pharmacology of newer insulin analogues. We also describe the novel technologies available and the considerations that need to be made by anaesthetists, surgeons, and other members of the perioperative team when looking after someone with diabetes mellitus on these insulins, or using these devices, to ensure safe care and the avoidance of complications.

**Editor's key points**

• Appropriate perioperative insulin manipulation allows for day or surgery admission, reduced incidence of dysglycaemia, and reduced length of stay. Individualisation of insulin management remains a challenge.
• This review summaries the pharmacology of different insulin types and how perioperative dose adjustments help to minimise dysglycaemia.
• Nonspecialists need to familiarise themselves with perioperative insulin manipulation, particularly with the imminent arrival of weekly basal insulins.

Diabetes mellitus (DM) is one of the most common metabolic diseases worldwide. It is characterised by hyperglycaemia, caused by defective insulin secretion, usually in type 2 DM (T2DM) failing to overcome decreased insulin sensitivity. The global prevalence of DM is ∼11% (828 million),^1^ and several types have been identified^2^: (1) type 1 DM (T1DM) results from pancreatic β-cell destruction by immune-mediated or idiopathic causes, leading to decreased or absent insulin secretion; it has a prevalence of ∼0.5%; (2) T2DM is caused by reduced sensitivity of the specific insulin receptor found on cell membranes and is characterised by insulin resistance; it has a prevalence of ∼8.5%; (3) diabetes may result from other, established aetiologies including genetic syndromes, monogenic disorders, or secondary diabetes as a result of pancreatic disease (e.g. cystic fibrosis), post pancreatectomy, infections, endocrinopathies, and medications (e.g. corticosteroids, HIV medications, or drugs used to treat mental health disorders); and (4) gestational DM is a form of hyperglycaemia that develops in a woman during pregnancy.

Diabetes represents a major healthcare burden. It causes increased morbidity and mortality from accelerated arteriopathy (both microvascular and macrovascular), deranged metabolic states, and predisposition to infections. Diabetes can complicate the peripartum period and can cause maternal and fetal harms.^3^ The association between cancer and diabetes is now well recognised,^4^^,^^5^ and there is increasing evidence that joint disease and osteoarthritis are associated with diabetes.^6^ Thus, although the prevalence of diabetes is ∼10% in the community, it is ∼25% in the inpatient and surgical patients, and it may be as high as 35% in the critical care patients, particularly in the cardiac or cardiac surgical patients.^7^ Diabetes is also associated with cognitive and functional disability, metabolic dysfunction-associated fatty/steatotic liver disease (MASLD/MAFLD), obstructive sleep apnoea, and depression.^4^ Therefore, optimising glycaemic control from the time of diagnosis is paramount to prevent or delay the onset of these multiple chronic complications.^8^

To mitigate the complications of diabetes, several therapeutic options are available that can be broadly characterised into pharmacological and non-pharmacological. This narrative review discusses the insulins that are available for the management of diabetes, and the pharmacological options that are available for managing diabetes with insulins in the perioperative period and within critical care. Furthermore, the risks associated with each option are discussed.

## Development of therapeutic insulin

In the late 19th century, it was identified that when the pancreas of a healthy dog was removed, the dog developed diabetes and died shortly after. It was postulated that this was owing to a missing chemical, later named insulin. In 1921, Banting and Best, who later received Nobel Prizes, extracted insulin from healthy dogs, and successfully treated pancreatectomised dogs.^9^^,^^10^

In January 1922, Leonard Thompson, a 14-yr-old boy dying as a result of T1DM in a Toronto hospital, became the first person to benefit from animal-derived insulin. After 1 yr, Eli Lilly introduced Iletin®, world's first commercially available insulin product for the treatment of diabetes; however, its drawbacks included short duration of action, multiple injections, high risk of hypoglycaemia, and allergic reactions. In 1936, Hans C. Hagedorn added protamine (a protein derived from salmon sperm), thus creating a longer-acting insulin with a better time action profile. After 10 more yr, neutral protamine Hagedorn (NPH) insulin, also called as isophane insulin, was developed by adding zinc to protamine insulin. This allowed the longer-acting insulin to be mixed with regular insulin in the same syringe. This mixed insulin improved glycaemic control and patient acceptability, allowing for twice-daily administration. NPH was subsequently used as the basal insulin to mirror physiological basal release.

Until 1982, animal-derived insulin was the only therapeutic option for T1DM, but its use was associated with the formation of anti-insulin antibodies leading to insulin resistance and lipoatrophy. In 1982, recombinant human insulin became available as either the regular and short-acting form (e.g. Humulin R®, also known as Humulin S® in the UK) or the intermediate-acting form NPH (isophane) insulin (e.g. Humulin N®, also known as Humulin I® in the UK) and rapidly replaced animal-derived insulin. However, it quickly became clear that the administration of a physiological hormone in an unphysiological way, that is s.c. rather than into the portal circulation, led to problems with dysglycaemia. The hope of mimicking physiological hepatic and peripheral insulin concentrations meant that insulins needed to be developed that had rapid s.c. absorption and peak effect, with short duration of action, and longer-acting insulins which had a prolonged action profile compared with human-derived isophane insulins, whilst at the same time avoiding the risk of hypoglycaemia.^11^^,^^12^

In an attempt to overcome these challenges, human insulin was modified, either by altering the amino acid sequence, adding free fatty acid chains to the insulin molecule, or other modifications to allow changes to the rate of absorption. This led to the development of the rapid-acting and long-acting analogue insulins. Table 1 shows a summary of modifications to insulin molecule in analogue and human insulins.

**Table 1:** Summary of modifications to insulin molecule in human and analogue insulins.^11^^,^^13^^,^^14^ NPH, neutral protamine Hagedorn.

Name of insulin	Protein sequence	Additional modifications	After s.c. injection	Speed of onset of action (min)	Time to reach maximal plasma concentration (h)	Duration of action (h)
	*Short-acting insulin*					
Human regular (soluble) insulin	Single insulin molecule consists of an A and B chain, connected by two disulphide bridges. Six molecules of insulin are positioned around a zinc ion to form a hexamer.	Nil	Hexamers slowly dissociate to dimers and monomers.	30	1.5–3.5	7–8
	*Rapid-acting insulins: first generation*					
Insulin lispro (standard)	Similar to human insulin, except that the amino acids at position 28 and 29 on the B chain are swapped over to lysine at position 28 and proline at 29.	Nil	Hexamers rapidly dissociate to dimers and monomers.	15	1–2	2–5
Insulin aspart (standard)	Similar to human insulin, except that the amino acid at position 28 is aspartate not proline.	Nil	Hexamers rapidly dissociate to dimers and monomers.	10–20	1–3	3–5
Insulin glulisine	Similar to human insulin, except lysine is substituted for valine at position 3 or the B chain and glutamate for lysine at position 29.	Nil	Hexamers rapidly dissociate to dimers and monomers.	10–20	0.5–1.5	4–6
	*Faster-acting analogues*					
Insulin lispro (fast-acting)	Identical to insulin lispro	Formulation contains citrate and treprostinil, a vasodilator.	Hexamers rapidly dissociate to dimers and monomers.	20	1–3	5–6
Insulin aspart (fast-acting)	Identical to insulin aspart	Formulation contains nicotinamide and L-arginine as excipients.	Hexamers rapidly dissociate to dimers and monomers.	5–15	1–3	3–5
	*Extended-acting insulins*					
NPH or zinc protamine	Single insulin molecule consists of an A and B chain, connected by two disulphide bridges. Six molecules of insulin are positioned around a zinc ion to form a hexamer.	Resuspended in zinc or protamine, respectively, which results in the formation of ‘conglomerates’ that prolongs duration of action.	Forms hexamer–protamine conglomerates.	60–120	4–12	20–24
	*First-generation analogues*					
Insulin detemir	Similar to human insulin, except for deletion of threonine at position 30 on the B chain.	Fatty acid moiety attached to the end of B chain.	Forms dihexamers that bind to albumin.	240–360	6–8	14–24
Insulin glargine	Similar to regular insulin, but glycine at position 21 of A chain and prolongation of B chain with two additional arginine residues.	Changes in amino acid alter the isoelectric point of the insulin.	Forms hexamer aggregates.	240–360	12	16–24
	*Second-generation analogues*					
Insulin degludec	Similar to human insulin, except for deletion of threonine at position 30 on the B chain.	Fatty acid moiety attached to the end of B chain.	Forms multihexamers that slowly dissociate and bind to albumin.	540	10	>42
	*Weekly insulins*					
Insulin icodec	Similar to human insulin, two peptide chains linked by a disulphide bridge.	A C20 fatty diacid-containing side chain has been added, and three amino acid substitutions.	The fatty diacid-containing side chain leads to strong, reversible binding.	N/A	Steady state reached in 3–4 weeks	∼200
Insulin efsitora alfa (basal insulin Fc – BIF) (not currently available)	Fusion protein combining a novel single-chain insulin variant together with human IgG2 Fc domain.	Human insulin receptor (IR) agonist fused to a human IgG2 fragment crystallisable (Fc) domain.	Binds to the neonatal Fc receptor to lengthen the time action profile.	N/A	Steady state reached in 6 weeks	∼400

In 1996, insulin lispro, the first of the recombinant short-acting analogues was marketed. By exchanging the amino acid lysine at position B29 and proline at position B28, faster dissociation absorption was achieved. Other short-acting analogues have subsequently been marketed (e.g. aspart and glulisine).^15^

NPH insulin had a role as basal insulin for ∼50 yr until insulin glargine was approved in 2000. In insulin glargine, glycine replaced asparagine at position A21, and an arginine molecule was added at position B31 and B32 to flatten the peak and provide a longer-acting profile. These modifications in amino acid sequences enhanced chemical stability, thus enabling slow release and long duration of action. Other long-acting analogues have subsequently been developed, namely insulin glargine U-300 (three-fold concentrated), insulin detemir, and insulin degludec. These newer long-acting insulin analogues have a more physiological basal profile and a lower risk of hypoglycaemia than the first-generation long-acting insulin glargine.^15^ Newer, once-weekly insulins are also now available or about to enter the market (e.g. insulin efsitora and insulin icodec).^13^ There is some evidence that using the extra-long duration insulins may have lower within-day variability and similar or marginally higher rates of hypoglycaemia.^13^^,^^16^, ^17^, ^18^ This is important because of the inpatient and outpatient harms associated with hypoglycaemia.^19^^,^^20^

Over the recent years, the patent of several insulin molecules has ended allowing the development of biosimilar insulins.^21^ Biosimilar insulins are almost identical copies of the original molecule and thus have very similar glucose-lowering properties to the original but may be produced in a different way. Insulin aspart (as Trurapi®), insulin lispro (as Admelog®) insulin glargine (as Semglee® and Abasaglar®) all have biosimilar equivalents and are available at lower cost than the original insulins. These medications are increasingly used because of their cost savings.

**Fig 1:**
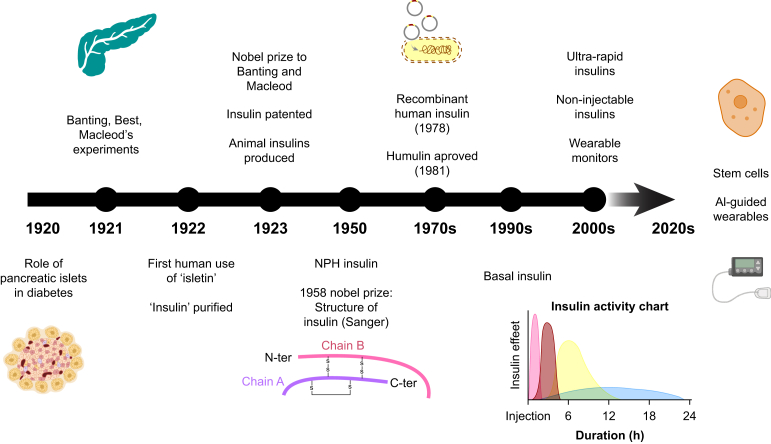
The history of the development of therapeutic insulin. The early insulin types administered to humans in the 1920s were bovine or porcine. AI, artificial intelligence; NPH, neutral protamine Hagedorn.

The history of the development of therapeutic insulin is summarised in Figure 1.

## Types of insulin

Insulins are increasingly classified according to pharmacokinetic principles rather than the source (animal derived *vs* human derived). Broadly speaking there are five different types of insulin: (1) rapid-acting insulin analogues, such as insulin aspart (Novorapid®, Fiasp®, or Trurapi®), insulin lispro (Humalog®, Lyumjev®, or Admelog®) or insulin glulisine (Apidra®); (2) regular soluble human insulin, also known as short-acting soluble human insulin (e.g. Actrapid® or Humulin S®); (3) intermediate-acting insulin, such as NPH (e.g. Insulatard® or Humulin I®); (4) long-acting insulin analogues, such as insulin glargine (Lantus®, Semglee®, or Abasaglar®), insulin detemir (Levemir®), insulin degludec (Tresiba®); and (5) ultralong-acting insulin analogues, such as insulin icodec (Awiqli®).

Each insulin type has its own pharmacokinetic profile, which defines its clinical use (Table 1).

In addition, there are also mixed insulins (also known as biphasic or premixed insulins). These are intermediate insulins premixed with either rapid-acting insulin analogues or soluble human insulin.

## Mechanism of action of insulin

Insulin is a peptide anabolic hormone produced by β-cells of the islets of Langerhans within the pancreas. It has several effects depending on plasma concentration. At the lowest concentrations it switches off ketone production, then stops hepatic gluconeogenesis (i.e. prevents glucose rising). At higher plasma concentrations it then allows for cellular glucose uptake, then glycogen synthesis, and is finally an anabolic hormone.^22^ Insulin resistance will lead to reduced insulin sensitivity and altered metabolic states, whereas absolute absence of insulin will cause a catabolic state and promote ketogenesis.^23^

## The international unit of insulin

During 1920s and the development of insulin, it was realised that there needed to be a system of documenting the potency of the insulin preparation. The mass was not appropriate as weighing scales were not accurate enough and the impurities could affect the weight too. The early definition of a unit of insulin was based on the amount of insulin required to produce a hypoglycaemic convulsion in a 2 kg rabbit.

With modern scientific methods, one international unit of unmodified human insulin is now defined as 0.0347 mg of anhydrous insulin.^24^

The relevance of this is that when prescribing insulin, it is imperative to write the word ‘units’ in full, and not to use any abbreviations, because otherwise people may misread the prescription and give an incorrect amount of insulin (i.e. ‘U’ being interpreted as ‘0’). In addition, only syringes that are designated insulin syringes should be used. To help mitigate the risk of incorrect dosing, dedicated insulin pens are now increasingly advocated.^25^^,^^26^

## Insulin regimens for people with type 1 diabetes mellitus in the community

People with T1DM must be administered exogenous insulin. Currently, other than amylin analogues in the USA, no other drug is licensed for use for treating T1DM—and then only as an adjunct to insulin. Amylin is a neuroendocrine hormone co-secreted by the pancreatic β-cell that has been shown to improve glycaemic control by slowing gastric emptying and promoting satiety.^27^ People with T1DM may be on different regimens; whilst in parts of the world where technology is available, the use of insulin pumps is very common (see next section), many people are on a basal-bolus regimen, that is, a dose of ultrarapid- or rapid-acting insulin at the time of a meal, with a longer-acting, basal insulin being given once or twice daily. This allows more flexibility at mealtimes and different carbohydrate content compared with the twice-daily mixed insulin regimens. A minority of people remain on twice-daily mixed insulin (e.g. Humalog mix 50®, or Novomix 30®), where the number represents the proportion of the mixture that is the short-acting insulin. They are most often given at breakfast and with the evening meal. However, occasionally these insulins are given three times a day, with the extra dose being given at lunch time.

## Continuous subcutaneous insulin infusions/insulin pumps

Increasingly, people with T1DM are on continuous s.c. insulin infusions (CSII), or insulin pumps. These devices deliver an infusion of rapid-acting insulin (e.g. insulin aspart) continuously under the skin. The hourly rates of infusion can be altered by changing the settings on the infusion pump. In the UK, they are now the standard of care for children and young people with T1DM, but increasingly so for adults as well. There is increasing use for those with T2DM using CSII, particularly in the USA. Although the rate of background insulin delivery can be set for each hour, the individual still needs to ‘announce’ to the device when carbohydrate is being eaten to allow a bolus dose to be given. S.C. short-acting insulin has a s.c. residence half-life of ∼45 min, but a circulating half-life of just 5 min. Thus, s.c. pumps can only be disconnected for up to 1 h, after which ketosis may develop. Such decompensation is very rapid if i.v. infusions are disconnected. A correction bolus of insulin may need to be given only if the disconnection time has been for longer than 1 h, not otherwise.

## Hybrid closed loop technology

Hybrid closed loops (HCLs) have also now been introduced into clinical practice.^28^ These devices comprise a continuous glucose sensor that measures interstitial fluid glucose linked by radiofrequency to an insulin pump. Similar to CSII, the HCL system involves continuous s.c. infusion of rapid-acting insulin analogues. An algorithm (usually on an app) is used to ensure an appropriate delivery of the rapid-acting insulin depending on the glucose concentration to replicate basal secretion by the pancreas. It is ‘hybrid’, as with CSII, because the individual still needs to ‘announce’ to the device when carbohydrate is being eaten. This enables the pump to deliver a bolus of insulin at the appropriate time. In due course, a ‘fully’ closed loop system is likely to be introduced where such announcement will no longer be necessary, and the device will detect the increase in postprandial glucose automatically and adjust the insulin infusion rate accordingly.

In 2023, the UK National Institute for Health and Care Excellence recommended that HCL technology should be rolled-out in a phased implementation to most people with T1DM.^28^

## Insulin regimens for people with type 2 diabetes mellitus in the community

In T2DM, insulin is frequently prescribed to be used alongside most other non-insulin diabetes medicines, including (but not limited to) sodium–glucose cotransporter 2 (SGLT-2) inhibitors, oral and s.c. glucagon-like peptide 1 or glucagon-like peptide 1/glucose-dependent insulinotropic polypeptide receptor agonists. For those with T2DM, there are various insulin regimens available, and there is a recognised step-wise escalation in the regimens. When glycaemic control is suboptimal (i.e. >69 mmol mol^−1^ [8.5%]) on maximal appropriate non-insulin medication, then a once-daily basal long-acting insulin (e.g. insulin glargine) may be added. If that is not appropriate, or insufficient to enable optimal glycaemic control, then, as with T1DM, a twice-daily or thrice-daily mixed insulin regimen, or even a basal-bolus regimen may be used. Currently, in the UK, the use of insulin pumps or HCLs are not recommended for those with T2DM.

Regimens are tailored to an individual's needs. With the data showing that the older one is at the time of diagnosis, the less years of life are lost as a result of diabetes, it may not be the glycaemic control that should be prioritised as the individual with diabetes gets older, but rather the avoidance of symptomatic hypoglycaemia and hyperglycaemia.^8^^,^^29^ Care is taken, particularly for those who have increased frailty or other comorbidities that increase the immediate risk of harms from hypoglycaemia rather than the theoretical risk of microvascular or macrovascular complications several years after the diagnosis of diabetes.

## Developments of perioperative insulin regimens

Before the introduction of detailed perioperative insulin regimens, surgery on people with diabetes carried a mortality risk of 3.7–13.2%. Death was attributed to myocardial disease, infection, and overt diabetic ketoacidosis (DKA). In 1979, Alberti and Thomas^30^ described a simple i.v. regimen that facilitated glycaemic control and prevented ketoacidosis. This regimen revolutionised the perioperative care and outcome of patients with diabetes. His scheme still dictates the principles for the perioperative pharmacological management of diabetes: (1) insulin administration is required to prevent lipolysis and subsequent DKA in patients with T1DM; (2) the ideal blood glucose zone for surgical patients on insulin therapy is approximately 5–10 mM; (3) hypoglycaemia especially in the anaesthetised patient with diabetes should be avoided; and (4) hyperglycaemia in surgical patients predisposes to infectious and non-infectious complications and should be avoided.

By 1985, the Alberti regimen (also known as the glucose–insulin–potassium [GIK] regimen) had become an established method for maintaining glycaemic control and metabolic stability; however, it was labour intensive.^31^ With increased availability of i.v. pumps, the GIK regimen was replaced in many centres by the variable rate i.v. insulin infusion (VRIII).^32^ This comprises two separate infusions, an infusion of glucose and potassium via one pump (administered at a constant rate), and a separate infusion of soluble insulin via a second pump titrated to the capillary blood-plasma glucose (CBG).

Although VRIII in theory should maintain glycaemic control and metabolic stability, in practice it is associated with a number of potential harms (Table 2). Furthermore, its use precludes day surgery. With the advent of insulin analogues, it was realised that perioperative administration of these insulins could lead to the avoidance of i.v. insulin infusions (i.e. GIK and VRIII).^34^ Thus, the inherent risks and complications of i.v. insulin could be averted (Table 2). Consequently, where possible, perioperative manipulation of the patient's normal insulin is increasingly recommended, provided certain criteria are met (Table 3).^35^^,^^36^

**Table 2:** Causes of physiological harms associated with the use of the variable rate i.v. insulin infusion (VRIII).^33^ CBG, capillary blood-plasma glucose; T1DM, type 1 DM.

Metabolic issue	Cause
Hypoglycaemia	• Wrong programming • Lack of one-way antisiphon valves in the setup • Insufficient measurement of CBG resulting in poor titration • Inappropriate titration scale being used • Titration scales that promote tight glycaemic control (e.g. CBG 4.0–6.0 mM) resulting in inappropriately high infusions of insulin • Acting on erroneously high blood glucose caused by glucose-containing solution in arterial line • Delays because of lack of staff • Inadvertent cessation of the substrate but with continuation of the i.v. insulin infusion
Hyperglycaemia	• Insufficient measurement of CBG resulting in poor titration • Delays because of lack of staff • Inappropriate titration scale being used
Diabetic ketoacidosis	• Delayed commencement • Discontinuation in a patient with T1DM without prior administration of appropriate basal insulin
Hyponatraemia	• Hypotonic solutions and insufficient sodium in the substrate fluid
Hypokalaemia	• Insufficient potassium in the substrate fluid

**Table 3:** Criteria for the surgical patient with diabetes mellitus to have their diabetes controlled by manipulation of normal medication.^33^

Patient factors	Surgical factors	Institutional factors
• Normally adequate glycaemic control (in the UK defined as an HbA1c <69 mmol mol^−1^ or <8.5%) • Stable and non-septic • Ability to understand instructions	• Short starvation period • Only one missed meal • Low expectation of postoperative starvation/ileus	• Ability to reliably give the patient a time for surgery to ensure that the patient will only miss one meal • Ability to prioritise the patient on the operating list • Ability for a trained member of staff to discuss perioperative manipulation of drugs with the patient, ensuring that the patient is able to follow the instructions • Ability to perform safe discharge of the surgical patient with diabetes and ensure that the patient understands when to seek medical advice (i.e. follow ‘sick day rules’)

Despite evidence of harm, some surgical centres persevere with intermittent s.c. boluses of soluble insulin. In the North American literature, this practice is called ‘sliding scale’ insulin, and although it is simple, it is not effective at maintaining glycaemic or metabolic control.^37^ As with the use of VRIII in the UK, the use of concurrent long-acting s.c. insulin is preferred to allow for an easier transition to s.c. insulin.^38^

## Perioperative insulin regimens

### Alberti, or glucose–insulin–potassium, regimen

The Alberti or GIK regimen involves administration of 500 ml of 10% glucose, with 1 g potassium chloride, and 10 units of soluble insulin to run at 100 ml h^−1^. If left unchecked, this can lead to fluid overload and hyponatraemia. The regimen demands the establishment of a new bag of 10% glucose with an altered amount of insulin should the blood glucose fall out of the range of 5–10 mM.^30^ Potassium is required to prevent hypokalaemia caused by the insulin stimulating the Na^+^/K^+^-ATPase protein pump in the cell membrane. In many centres, the use of the GIK regimen has been superseded by the VRIII regimen described below, but there is evidence it continues to be used.^34^^,^^39^

### Variable rate intravenous insulin infusion

The VRIII appears to have been adopted into clinical practice to overcome the shortcomings of the GIK regimen. There is an absence of literature detailing its introduction and demonstrating its safety and efficacy.

The VRIII consists of two components: the soluble i.v. insulin infusion and the fixed rate substrate solution containing glucose and potassium. Initially this regimen in the UK was known as the ‘sliding scale’; however, to avoid confusion with the North American definition of sliding scale insulin, the two separate infusions are known as VRIII.

The VRIII needs meticulous 1–2 hourly measurements of the capillary glucose, and subsequent adjustments to the rate of the insulin infusion, otherwise dangerous hypoglycaemia may result.

Initial descriptions of the VRIII only promoted the use of potassium in glucose-containing solutions. Although not all glucose-containing solutions are hypotonic, it is now recognised that the use of hypotonic solutions will cause hyponatraemia and detailed protocols need to be adopted to promote the safe use.^35^^,^^36^ Even dedicated nursing input cannot prevent harms.^40^ Thus, tight blood glucose control while on i.v. insulin is increasingly difficult to advocate, but glucose concentrations of <10 mM are associated with lower risk of mortality.^41^ However, whether this is causation or association remains to be elucidated.

As delays in establishing the VRIII and taking down the VRIII may cause an absence of insulin, and precipitate lipolysis and ketogenesis, the administration of a modified dose of the patient's basal insulin is often administered concurrently with the VRIII.

### ‘Sliding scale’ insulin

Sliding scale insulin regimes involve s.c. administration of soluble insulin when hyperglycaemia is detected. As this regimen is reactive to elevated blood sugars, ‘sliding scale’ insulin does not provide good glycaemic control.^42^ When compared with basal-bolus regimes, it is associated with higher mean blood glucose concentrations and a higher incidence of hyperglycaemic events.^42^, ^43^, ^44^ There is some evidence that it associated with greater morbidity.^43^ Consequently, ‘sliding scale’ insulin for perioperative glycaemic control is increasingly discouraged.^37^

### Correction doses

The concept of correction doses is to provide a ‘one-off’ dose of a fast-acting insulin to correct a single incidence of elevated blood sugar, in a patient on background treatment. For adult patients, UK guidance suggests giving a dose of 2–6 units of s.c. rapid-acting, analogue insulin for blood glucose concentrations >12 mM with no evidence of DKA or hyperosmolar hyperglycaemic state (HHS). This is for people with T1DM, where 1 unit will generally reduce the CBG by 3 mM. Wherever possible, people with T1DM should be asked what their own correction factor is. People with T2DM should be asked what they normally do, but if this is unknown or for those who are not previously treated with insulin, they should be given 0.1 units kg^−1^.^35^

Repeated administration of correction doses would be akin to the sliding scale insulin regimen and therefore cannot be advocated. If an individual requires more than two correction doses, they should be reviewed by the medical team, and the need for an increase in the pumped or basal insulin dose considered.

### Basal-only insulin regimen for inpatient glycaemic control

Basal-only regimes for inpatient glycaemic control are essentially a form of modification of the dose of the patient's normal long-acting insulin. Administration of ∼75–80% of the patient's usual insulin glargine dose the night before surgery is associated with optimal glycaemic control and minimal risk of hypoglycaemia.^45^^,^^46^ Basal insulin also controls hepatic and adipose tissue metabolism in the fasted state.

There is some evidence that using long-acting insulins with an extra-long duration of action may have lower within-day variability and reduced rates of hypoglycaemia.^47^

### Basal-bolus insulin regimen for inpatient glycaemic control

Basal-bolus insulin for inpatient glycaemic control replicates the principles of basal-bolus insulin in the community. Patients take once-a-day long-acting insulin and then take a rapid-acting insulin with meals (if eating).

The original multicentre basal-bolus study described recruiting surgical patients with T2DM, who were either on oral glucose-lowering agents or minimal amounts of insulin.^43^ They were randomised to either a basal-bolus insulin therapy or sliding scale insulin. The dose of the basal insulin glargine was 0.25 units kg^−1^ if fit and well, and 0.15 units kg^−1^ if comorbidities present. The bolus dose of rapid-acting insulin glulisine was to be given with meals and was either 0.08 units kg^−1^ or 0.05 units kg^−1^ if frail.^43^ The basal-bolus regimen improved glycaemic control and reduced hospital complications compared with sliding scale insulin in general surgery patients.

Although this strategy is clearly effective, the methodology is very labour intensive, and this may hinder widespread adoption and replication of the encouraging results.^48^

### Basal plus correction insulin regimen for inpatient glycaemic control

The basal plus correction insulin regimen was designed to overcome the apparent complexity of the basal-bolus insulin regimen for inpatient glycaemic control. It involves the administration of once-a-day basal insulin and the administration of correction doses of a rapid-acting insulin to rectify hyperglycaemia.

The original multicentre study described recruiting surgical and medical patients with T2DM, who were either on oral glucose-lowering agents or minimal amounts of insulin. Patients were enrolled into a multicentre three-armed study. The three cohorts were basal plus correction, basal bolus, and ‘sliding scale’ insulin. The basal plus correction regimen was demonstrated to provide better glycaemic control and fewer hypoglycaemic episodes than the other two regimes. Again, ‘sliding scale’ insulin was found to provide the worst glycaemic control.^49^

### Modification of usual insulin regimen

Despite having a limited evidence base,^50^ modification of the patient's usual insulin regimen is recommended by several UK and international organisations.^35^^,^^36^^,^^51^^,^^52^

Preoperative assessment is essential to gauge suitability for modification (Table 3), and to give the patient or their care provider detailed guidance on the alterations to the patient's normal medicines.

For patients on basal insulins, the evidence-based advice is to take 75–80% of the usual long-acting insulin the night before surgery.

For patients on basal-bolus regimes, the advice is to take 75–80% of the usual long-acting insulin the night before surgery, and to omit the preprandial dose for the omitted meal.

For patients on twice-daily mixed regimes, the general advice is to take the evening dose before surgery as usual, and then take, with caution, 50% of the morning dose.

It is a prerequisite that these regimes are only used if the patient will have one missed meal. If the patient develops postoperative nausea and vomiting, or an ileus, an i.v. insulin infusion should be commenced, with glucose and potassium supplementation as needed.

Previously this regimen was reserved for elective patients, but with the advent of day surgery trauma lists, it is now recognised that this strategy can be used for emergency surgical patients who meet the criteria.

### Continuation of the continuous subcutaneous insulin infusion/insulin pump

Continuation of the CSII is now well described in the literature, especially during the peripartum period^53^ despite the pump manufacturers mandating that their equipment should not be used near diathermy.^54^

For safe use of the CSII in theatre, strict selection and guidelines must be adhered to.^55^^,^^56^ In addition to the criteria discussed in Table 3, the patient should be seen by a specialist diabetes nurse before surgery and be given detailed advice. The patient should be instructed to use a polytetrafluoroethylene (PTFE) cannula rather than metal and to site the cannula away from the site of surgery. The patient should also be advised to aim for a CBG of 6–10 mM. This generally means a minor reduction in the basal infusion rate, and the omission of the preprandial bolus dose. In addition, it is advised that shared decision-making is undertaken so that the patient is involved in the decision to continue the pump, or to commence the alternative of multiple-dose insulin or VRIII.

### Continuation of hybrid closed loop technology

The HCL systems link continuous glucose monitoring (CGM) with insulin pump technology to monitor blood glucose and automatically adjust the amount of insulin given through a pump to people with T1DM. As CGM measures interstitial glucose concentrations rather than blood glucose concentrations, there is a lag between CGM and CBG, and thus the accuracy of the CGM sensor cannot be relied upon in the hospitalised patient, particularly if there is poor peripheral perfusion. Furthermore, the accuracy of the CGM sensor can be affected by pressure, electromagnetic interference, or medicines (e.g. paracetamol).^57^, ^58^, ^59^ In addition, the CGM sensor should be in place for at least 24–48 h and not approaching the time it needs to be changed. Furthermore, it should not need calibrating.

There have been case reports and case series of people with diabetes undergoing surgery with HCL,^59^ and there are now some rudimentary guidelines and expert consensus statements providing suggestions and recommendations on the perioperative use^57^^,^^58^; however, more experience is needed before it can be routinely recommended.^55^^,^^56^ Practices around the world may be different, particularly if the duration of surgery is due to be short.

For patients with HCL, the current options are: (1) discontinuing and disconnecting the pump and establishing a VRIII; (2) converting the pump into manual mode and relying on CBGs; and (3) continuation of the HCL but with close coordination between the diabetes and the anaesthetic teams and with regular checking of CBG.

Once HCL technology is more widely used and perioperative teams gain experience, provided certain criteria are met (Table 4), perioperative continuation of the HCL may be routinely advocated.

**Table 4:** Criteria for the surgical patient on hybrid closed loop (HCL) technology to have their diabetes mellitus managed with continuation of the HCL. CBG, capillary blood glucose; CGM, continuous glucose monitor(ing); DKA, diabetic ketoacidosis; HCL, hybrid closed loop; POC, point of care; PTFE, polytetrafluoroethylene.

Patient factors	Anaesthetic factors	Surgical factors	Institutional factors
• Normally adequate glycaemic control (as defined by HbA1c <69 mmol mol^−1^ or <8.5%) • Stable and non-septic • Physiologically stable with good tissue perfusion, and no evidence of sepsis or DKA • Ability for the person with diabetes/carers/diabetes team to resume responsibility for HCL technology in the immediate postoperative period	• Ability to perform CBG at 30-min intervals • Ability to minimise risk of disconnection of pump and CGM • Recognition that certain drugs will cause the CGM to misread • Contingency plan if HCL technology fails and becomes unsafe	• Short starvation period • Only one missed meal • Low expectation of postoperative starvation/ileus • Planned operation has no major fluid shifts • Ability to site CGM from pressure and the diathermy arc • Ability to site insulin pump away from diathermy arc and use bipolar diathermy if possible • Ability to protect the devices from electromagnetic interference (no magnetic resonance imaging)	• Ability to perform shared decision-making to discuss risks and benefits of the options • Ability to reliably give the patient a time for surgery to ensure that the patient will only miss one meal • Ability to prioritise the patient on the operating list • Ability for a trained member of staff to discuss perioperative manipulation of the HCL technology with the patient, ensuring that the patient is able to follow instructions • Ability for a PTFE needle to be sourced • Ability to set the glucose target for a temporary target above normal • Ability to perform safe discharge of the surgical patient with diabetes and ensure that the patient understands when to seek medical advice (i.e. follow ‘sick day rules’)

## Management of insulin in patients with dysglycaemia in the intensive care unit

In the critically ill patient with either diabetes or stress hyperglycaemia, current guidance advocates the use of i.v. infusion of short-acting soluble insulin (Actrapid® or Humulin® S).^60^ Most commonly, this will be in the form of a VRIII. The insulin dose/infusion rate is titrated to the blood glucose, which is measured at regular intervals. This recommendation is predominantly based on observation of a greater time in glycaemic range and more predictable dosing when using i.v. rather than s.c. agents, whose absorption could be impacted by critical illness and its treatment (e.g. catecholamines and vasopressors).^60^

No universal protocol for i.v. insulin therapy exists although the core components of any such protocol have been outlined by other authors.^60^ For patients with T1DM, i.v. insulin therapy must occur alongside a substrate infusion (classically a glucose solution containing potassium). However, in critical illness where fluid overload is a major risk, nasogastric/tube feeding or total parenteral nutrition may be used as a substrate in certain clinical situations.^61^

A fixed rate i.v. insulin infusion (FRIII) of short-acting soluble insulin is recommended for the management of certain diabetes-related emergencies including DKA, SGLT-2 inhibitor-induced euglycaemic ketoacidosis, and HHS.

In these circumstances, FRIII at a rate of 0.1 units kg^−1^ h^−1^ is advised.^62^^,^^63^ In order to prevent hypoglycaemia, it is advised to run 10% glucose concurrently once the blood glucose is <14 mM and to consider reducing insulin infusion to 0.05 units kg^−1^ h^−1^. Furthermore, preadmission long-acting insulin analogues should be continued.^38^

After acute illness, a transition between i.v. and s.c. agents is required, especially in those taking insulin before admission.^64^ If VRIII or FRIII has been used, then continuation of long-acting insulin/basal insulin, especially for patients with T1DM, should be considered and is increasingly advocated. However, the dose should be reduced. Post-transition, hyperglycaemia rates may be increased, although hypoglycaemic events are less common.^65^ Adjustment is not standardised although studies have reported using total s.c. doses amounting to 50–70% of the total daily i.v. insulin dose.^65^, ^66^, ^67^ Usual precautions in transitioning from i.v. insulin should also be adhered to, including the administration of background insulin before discontinuing i.v. insulin.^38^

## Ensuring safe use of insulin for diabetes mellitus in the perioperative period

The management of diabetes during the perioperative period should begin as soon as the decision to proceed with a surgical procedure is made. For elective procedures, ideally this should begin at the time of primary care referral and be an ongoing process in the outpatient clinic and be continued in the preoperative assessment clinic. This provides opportunities to allow optimisation of diabetes and agreement on a plan for diabetes medicines during the perioperative period.^68^ Together with appropriate clinical resources, diabetes inpatient specialist nurses (DISNs) can coordinate individualised perioperative care for people with diabetes and improve safety of medicines used in the treatment of perioperative diabetes.^35^

Pharmacy teams have an integral role in ensuring safe administration of medicines during the perioperative period.^69^ Increasingly, they are an integral part of preassessment clinics to ensure reconciliation of medicines before admission to reduce medication errors.^70^ On admission, patients should bring their diabetes medicines to hospital. This avoids omitting doses and ensures that they can continue to use medicines that they are familiar with, particularly if self-administration is intended.^35^ Pharmacists also facilitate medication reconciliation at the time of hospital discharge to ensure proper dosing and medication follow-up.

Insulin is a high-risk medicine and errors in the administration of insulin by clinical staff has contributed to numerous patient safety incidents that may be severe and can cause death.^57^^,^^71^^,^^72^ In the UK, a National Patient Safety Agency Rapid Response Report in 2010 made recommendations to healthcare organisations to reduce the risk. These include: (1) using an insulin syringe or commercial insulin pen device for measuring and administering all bolus insulin doses; (2) use of the term ‘units’ in all contexts and never use abbreviations, such as ‘U’ or ‘IU’; and (3) people undergoing elective surgery should be advised to brings supplies of their usual insulins.

## Technological solutions

More routine adoption of electronic health records (EHRs) and electronic prescribing and medicines administration (EPMA) systems offers opportunities for technological solutions to improve perioperative care for people prescribed medicines for DM. Clinical decision support (CDS) tools combine EHR data about an individual together with population statistics and best-practice guidelines to individualise recommendations. These CDS tools can be embedded into current EHR and EPMA workflows to support clinical decision-making. CDS tools using EHR data have been used successfully to reduce hyperglycaemic events and inappropriate insulin use among inpatients with diabetes. However, hypoglycaemic episodes or impending hypoglycaemia were unrelated to use of the CDS tool.^73^ Other uses for CDS tools include setting glycaemic targets, providing haemoglobin A1C reminders, guiding weight-based dosing, and matching an insulin regimen to nutritional profile.^74^

An alternative approach is a virtual glucose management service (vGMS) that uses EHR to generate a report of out-of-range glucose values to be reviewed by diabetes specialists, who then remotely review and make recommendations.^75^ After the introduction of vGMS, the proportion of people with hyperglycaemic and hypoglycaemia episodes reduced by more than one-third.

Discharge should be planned proactively and started early in the surgical pathway. Timely communication with primary care is required, particularly if there have been changes to medicines for diabetes or issues with glycaemic control during the hospital stay.^35^ The patient should be provided with verbal and written advice. This should include how to manage their diabetes during their recovery including sick day rules, changes to prescribed medicines, and who to contact for advice regarding diabetes management and postsurgical issues.

## Conclusions

Since the publication of Alberti's seminal 1979 paper advocating the use of the GIK regimen, huge changes in the perioperative management of diabetes have occurred that have enabled patients with diabetes to have safer surgery (including day surgery) and shorter length of stays. These advances have only been possible with the introduction of novel insulin medicines and the increasing use of technology. Underpinning these advances is the collaborative interdisciplinary work involving diabetologists, anaesthetists, nurses, dieticians, and pharmacists. This work has elucidated the safe perioperative use of the medicines that maintain blood sugars in a safe optimal zone while preventing harm from diabetes medicines. This review has discussed the current state of the safe use of insulin medicines during surgery and critical care admission; however, it is expected that further medications and drug delivery systems will be developed, and thus strategies will continue to evolve. Future researchers and perioperative teams will need to elucidate the precise required perioperative modifications for the new technology and medicines; however, the underlying principles of maintaining the glucose concentration in a safe range, preventing hypoglycaemic or hyperglycaemic crisis, and preventing adverse drug events will remain.

## Authors’ contributions

Conception: KD, NL

Writing of all drafts of the manuscript: KD, NL, DS, CF, SLT, RDG

Manuscript review and approval of the final version: all authors

## Declaration of interest

The authors declare that they have no conflicts of interest.
